# Better Than Nothing? Limitations of the Prediction Tool SecretomeP in the Search for Leaderless Secretory Proteins (LSPs) in Plants

**DOI:** 10.3389/fpls.2016.01451

**Published:** 2016-09-27

**Authors:** Andrew Lonsdale, Melissa J. Davis, Monika S. Doblin, Antony Bacic

**Affiliations:** ^1^ARC Centre of Excellence in Plant Cell Walls, School of BioSciences, The University of MelbourneParkville, VIC, Australia; ^2^The Walter and Eliza Hall Institute of Medical ResearchParkville, VIC, Australia; ^3^Department of Biochemistry and Molecular Biology, Bio21 Molecular Science and Biotechnology Institute, The University of MelbourneParkville, VIC, Australia

**Keywords:** protein localisation prediction, secretome, unconventional protein secretion, leaderless secretory protein, plant cell wall, SecretomeP

## Abstract

In proteomic analyses of the plant secretome, the presence of putative leaderless secretory proteins (LSPs) is difficult to confirm due to the possibility of contamination from other sub-cellular compartments. In the absence of a plant-specific tool for predicting LSPs, the mammalian-trained SecretomeP has been applied to plant proteins in multiple studies to identify the most likely LSPs. This study investigates the effectiveness of using SecretomeP on plant proteins, identifies its limitations and provides a benchmark for its use. In the absence of experimentally verified LSPs we exploit the common-feature hypothesis behind SecretomeP and use known classically secreted proteins (CSPs) of plants as a proxy to evaluate its accuracy. We show that, contrary to the common-feature hypothesis, plant CSPs are a poor proxy for evaluating LSP detection due to variation in the SecretomeP prediction scores when the signal peptide (SP) is modified. Removing the SP region from CSPs and comparing the predictive performance against non-secretory proteins indicates that commonly used threshold scores of 0.5 and 0.6 result in false-positive rates in excess of 0.3 when applied to plants proteins. Setting the false-positive rate to 0.05, consistent with the original mammalian performance of SecretomeP, yields only a marginally higher true positive rate compared to false positives. Therefore the use of SecretomeP on plant proteins is not recommended. This study investigates the trade-offs of using SecretomeP on plant proteins and provides insights into predictive features for future development of plant-specific common-feature tools.

## Introduction

The plant cell wall, a complex extracellular matrix of carbohydrate and some protein, is one of the defining features of plant cells. Protein accounts for up to 10% of the dry weight of the primary cell wall (Doblin et al., 2010) yet despite being a minor component, cell wall proteins (CWPs) play important roles in plants. CWPs can function in the plant cell’s normal growth and developmental processes, as well as in response to biotic and abiotic stresses. They can act to modify other wall components thereby altering the structure and composition of the wall in response to internal and external stimuli. Separating individual plant cells within either a tissue or organ is an open continuous compartment called the apoplast, which also contains (glyco)proteins and, together with the cell wall, collectively refers to the extracellular space. The collection of proteins found in the extracellular space is called the secretome.

Proteins at the cell surface (plasma membrane/apoplast) are typically glycosylated and usually trafficked via the highly conserved eukaryotic process of classical protein secretion involving vectorial transport through the ER and Golgi apparatus. These classically secreted proteins (CSPs) are typically targeted to the ER by an N-terminal signal peptide (SP) where it is subsequently cleaved. However, not all proteins destined for the cell surface have a SP in their sequence and those lacking this feature are referred to as leaderless secretory proteins (LSPs; Krause et al., 2013). These types of proteins are secreted via a route called unconventional (i.e., non-classical) protein secretion (UPS). UPS is a term that covers the atypical cases of LSPs but also some secreted proteins containing a SP that are non-classically secreted, either via intermediate organelles or vesicular bodies (Ding et al., 2014; van de Meene et al., 2016).

In plant proteomic studies, it has been estimated that over half the detected secreted proteins lack a classical SP (Krause et al., 2013). However, due to the possibility of contamination either from already lysed cells (as occurs in cell suspension cultures; Miernyk et al., 2016) or from cells undergoing programmed cell death (*in planta*) or from other sub-cellular compartments during the protein extraction procedure, not all proteins lacking a SP in a secretome experiment are expected to be true LSPs (Rose and Lee, 2010; Albenne et al., 2013). Determining which plant proteins are candidate LSPs that have undergone UPS and which have been introduced as contamination is therefore a challenge (Rose and Lee, 2010; Albenne et al., 2013; Krause et al., 2013). Given the high likelihood of contamination during sub-cellular fractionation procedures, a separate and independent assessment is needed in order to conclude that such proteins are indeed located in the ECS and are legitimate components of the plant secretome. There are three broad assessment strategies that can be implemented for an *in silico* approach: (1) accept all proteins found, a strategy likely to have a high false-positive rate; (2) include only proteins with SPs and reject others, a strategy likely to discard most real examples of LSPs; or (3) balance true and false positives by including proteins with SPs and filter others based on a prediction of a protein being a LSP. Ultimately, there is a need to adopt experimental approaches, for example immuno-localisation to verify the *in silico* predictions, irrespective of their veracity.

SignalP (Petersen et al., 2011) is often used to identify SPs, yet bioinformatics tools to predict LSPs have only been developed for mammals and bacteria (summarized in **Table 1**). None were trained on plant proteins and the lack of plant-specific tools for predicting LSPs using the third strategy above is a recognized problem for filtering approaches (Agrawal et al., 2010; Ding et al., 2012; Albenne et al., 2013). However, this has not prevented the research community from using tools such as SecretomeP (Bendtsen et al., 2004, 2005) as a filter in proteomics experiments attempting to characterize the plant secretome. As the oldest and most highly cited tool, it is perhaps unsurprising that the plant proteomics community has adopted it when using a filtering strategy. It has been applied to studies in various plant species, for example *Arabidopsis thaliana* (e.g., Jamet et al., 2008; Ge et al., 2011), *Oryza sativa* (rice) (e.g., Song et al., 2011; Wang et al., 2012), *Helianthus annuus* (sunflower) (e.g., Pinedo et al., 2012) and the moss *Physcomitrella patens* (e.g., Lehtonen et al., 2014).

**Table 1:** Details of non-classically secreted protein (CSP) prediction programs, as of April 2016.

Prediction tool	Reference	Target^1^	Method^2^	Number of predictions^3^	Available^4^	Citations^5^
SecretomeP (v1)	Bendtsen et al., 2004	M	W, D	500 (W), - (D)	Yes	642
SecretomeP (v2)	Bendtsen et al., 2005	B	W	100	Yes	342
SecretP (v1)	Yu et al., 2010b	M	W	1	Error	17
SecretP(v2)	Yu et al., 2010a	M, B	W	1	Error	76
SecretP (v2.1)	Yu et al., 2013	G-	W	1	Error	3
SPRED	Kandaswamy et al., 2010	M	D	-	Yes	15
SRTpred	Garg and Raghava, 2008	M	W	1	Yes	35
Sec-GO	Huang, 2012	M, B	W	?	No	7
NClassG+	Restrepo-Montoya et al., 2011	G+	W	?	No	6

*^1^Target: (M)ammalian, (B)acteria, (G+) Gram-positive bacteria, (G-) Gram-negative bacteria. ^2^Method: (W)ebserver, (D)ownload. ^3^Number of sequences that can predicted in a single run, - indicating no limit. ^4^Available refers to web server or program availability at the published location. Error indicates where the program is available but not operational. ^5^Citation counts from Google Scholar (http://scholar.google.com) as of April 2016.*

SecretomeP was trained on mammalian and bacterial proteins and the lack of plant training data is of concern when applying it to plants, as was noted by Agrawal et al. (2010) when they recommend the inclusion of SecretomeP in a plant proteomic analysis workflow. Albenne et al. (2013) clearly point out the deficiencies of this approach given the program was not designed for plants, and although some studies include caveats on its suitability, the hypothesis behind SecretomeP is often overlooked. This hypothesis assumes that secreted proteins will share common properties, regardless of their mechanism of secretion. This approach was taken to overcome the lack of known mammalian LSPs available for use as a positive training dataset, and enabled the authors to train their method using CSPs. This also allowed for the few known LSPs to be used after training to test the accuracy of the tool. The authors of SecretomeP propose that common properties would be captured by removing the SP from the sequences of CSPs to generate training sequences. Proteins sharing these common features, but lacking a SP are then predicted to be LSPs.

**FIGURE 1:**
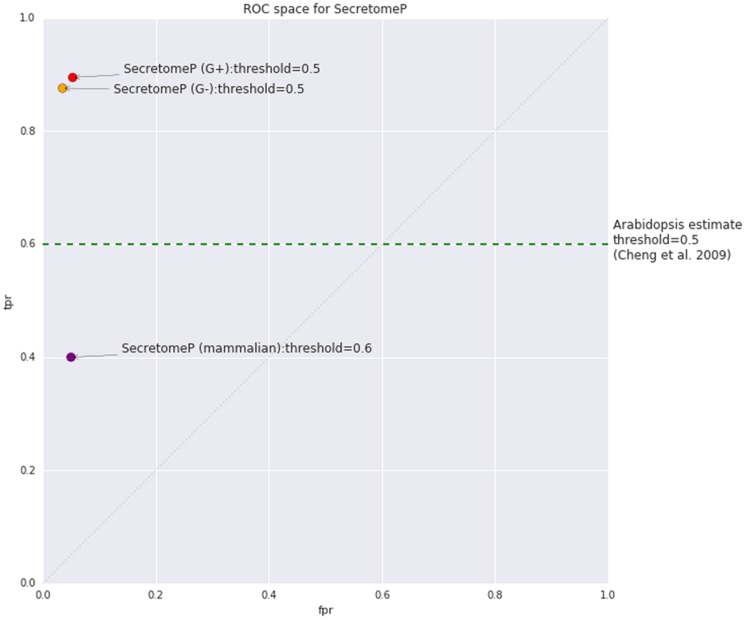
**Estimated true positive rate (TPR) and false positive rate (FPR) for SecretomeP as published for mammalian proteins (v1) based on internal cross-validation and bacterial proteins (v2) based on performance on classically secreted proteins (CSPs) from the SignalP 3.0 dataset.** The estimated TPR of SecretomeP on plants as stated by Cheng et al. (2009) was 0.6, with no FPR given. The random line diagonal represents equal TPR and FPR, equivalent to random selection of classes.

Using the mammalian version of SecretomeP as a tool for LSPs in plants assumes that any common features are also shared between mammalian and plant-secreted proteins. The software programs used to capture protein features in the mammalian versions of SecretomeP are listed in **Table 2**. It also implicitly assumes that the reported threshold and accuracy metrics of the mammalian version of SecretomeP will apply to plants. The threshold value used to generate positive predictions is of particular importance: SecretomeP outputs scores in a range from 0 to 1 to indicate increasing confidence that a protein is secreted. The trade-off between true and false positives for any given threshold is essential in applying the tool to experimental output. The authors of SecretomeP recommended using a threshold of 0.6 when using the method on mammalian proteins, giving a true-positive rate (TPR) of 0.40 and false-positive rate (FPR) of 0.05 (**Figure 1**). This score was derived from cross-validated sub-sets of the modified CSP training data. When applied to the 13 human LSPs at the time, 10 of these were observed to be predicted at this threshold (Bendtsen et al., 2004).

**Table 2:** Protein features used in SecretomeP and the programs it utilizes internally to calculate them.

Protein feature	Dependency	Reference
Number of atoms	-	-
Number of positively charged residues	-	-
Low-complexity regions	SEG	Wootton and Federhen, 1996
Sub-cellular localisation	PSORT II	Nakai and Horton, 1999
Transmembrane helices	TMHMM 2.0c	Krogh et al., 2001
Pro-peptide prediction	ProP 1.0c	Duckert et al., 2004

*- indicates feature calculated internally without any external dependency*.

The equivalent TPR and FPR when applied to plants has not previously been studied, although some accuracy values have been reported based on observation from proteomic studies. In an investigation of plant defense responses simulated by the application of salicylic acid, Cheng et al. (2009) stated that 60% of *Arabidopsis* LSPs were predicted to be secreted using a threshold of 0.5, based on the assumption that all the leaderless proteins they found were genuine. This value has also been reported in a more recent review (Agrawal et al., 2010) and both papers include the caveat that the program outputs should be used as indicative only due to the inconsistencies they found with predictions on SP-containing proteins. Even assuming no contaminants, the value only indicates how many true positives are correctly predicted. A TPR of 0.6 seems quite good, so its appearance in the literature could contribute to the continued use of this non-plant based tool on plant proteins. Interestingly, accepting at face value 0.6 as a TPR for SecretomeP on plant proteins this would indicate that the algorithm performs better on plant proteins than mammalian proteins (**Figure 1**). Whether this score should be extrapolated to be a measure of predictive performance on *Arabidopsis* under other conditions or on other plant species, however, is questionable. Even if all the proteins found are assumed to be true positives and extrapolated to other plant species and/or conditions, then it is only an estimate of the TPR and the FPR is unknown.

Establishing an optimal threshold and determining the performance metrics at that threshold is essential if the results of SecretomeP on plant proteins are to be useful. Ideally, evaluation would require positive (LSPs) and negative (non-secretory proteins) data to investigate the TPR and FPR for a given prediction threshold. However, since the issue is whether SecretomeP can accurately predict if a given plant protein is a LSP, the evidence that such a protein is unconventionally secreted needs to be obtained by means other than the output of the program under evaluation. There are a number of experimental approaches to determine the sub-cellular location of a protein, some relying on cell disruption and others on *in situ* microscopic approaches, assuming the relevant probes (e.g., antibodies) are available. The need for molecular approaches to validate putative LSPs is acknowledged in the literature (Albenne et al., 2013; Robinson et al., 2015; van de Meene et al., 2016) yet practically, in order to perform such a step, candidate proteins from a secretome will often need to be identified first. Since there are very few known plant LSPs and only one protein with experimental (biochemical) validation (Pinedo et al., 2012), a ‘gold standard’ to evaluate against does not currently exist. Although there are putative lists of LSPs [e.g., Ding et al. (2012)] the uncertainty surrounding their prediction means that their use as a training set would be rendered invalid.

Accepting the validity of the common-feature hypothesis used in SecretomeP provides a proxy positive data set, namely CSPs of the plant cell wall and apoplast. If this hypothesis is correct, we can estimate SecretomeP’s accuracy on plant LSPs by evaluating its performance on these known secreted proteins. This study investigates the trade-offs of using such an indirect tool on plant proteins and provides insights into predictive features for future development of plant-specific common-feature tools. If SecretomeP is to be used on plant secreted proteins, then its use needs to occur with knowledge of the true and false positive rates involved when the method is applied to plants.

## Materials and Methods

### Data Sources

*Arabidopsis* proteins were the focus of this study due to the availability of large databases of proteins with curated sub-cellular location. Protein sequences were obtained from WallProtDB (San-Clemente and Jamet, 2015) and the *Arabidopsis thaliana* sub-proteome reference (ASURE) (Hooper et al., 2014) databases. After excluding proteins less than 40 amino acids in length due to the SecretomeP cutoff, a total of 1983 WallProtDB and 975 ASURE proteins were included in analyses. WallProtDB proteins were used as positive CSP data. Various sub-sets of WallProtDB were also used as positive data to ensure conclusions from WallProtDB are not biased by its protein composition. Five sub-sets were made by restricting to maximum 30% sequence identity (278), *Arabidopsis* only (522), excluding *Arabidopsis* (1461), rice only (208) and *Brachypodium distachyon* only (358).

The ASURE database contains a smaller set of proteins representing each sub-cellular compartment in the broader SUBA database (Tanz et al., 2013) and as such is a mixture of proteins that are examples of positive, negative, and neutral for this study. The sub-set of CSPs labeled as Extracellular (42) was used as positive and sub-sets of non-secretory proteins located to the nucleus and/or cytosol (352) used as a negative. Sub-sets of other subcellular locations were also used as comparisons such as non-secretory proteins of the plastid (103) (Supplementary Table S1).

### Evaluation Data and Protein Sequence Modifications

The use of CSPs as a proxy for SecretomeP prediction of LSPs could be influenced by the components of CSPs rather than by any shared protein features. Removing the SP, in a similar way to the training of SecretomeP, removes this possible bias yet introduces a departure from actual protein data that could also influence the results. To ensure that the evaluation is based on common features rather than a feature unique to CSP such as the SP, the proxy data were modified in various ways to investigate the effect of SP changes on SecretomeP prediction scores.

**FIGURE 2:**
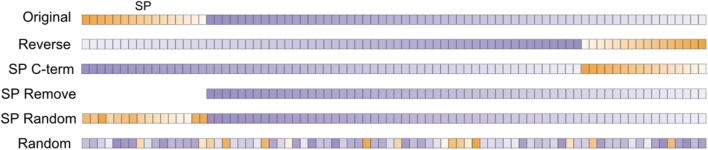
**After the SP region (orange) was identified using SignalP 4.1 for positive data or a fixed length of 30 amino acids for negative, five modifications were made to each original sequence in a dataset.** Reverse inverted the amino acid order of the entire sequence; SP C-term placed the SP at the C-terminus, leaving the sequence length unchanged; SP Remove excluded the SP region and shortened the sequence length. SP Random and Random involved random shuffling of the SP region and the entire coding sequence, respectively. The sequences were then submitted to SecretomeP. Orange, SP region; Purple, mature protein sequence.

Under the common-feature hypothesis, modifications to only the SP of CSPs should not influence the prediction score of SecretomeP. Five modifications were made under this assumption (**Figure 2**): (1) The SP was removed (SP Remove) but because this results in a shorter sequence, sequences were also modified to alter the SP without either changing the length of the original sequence or its amino acid composition. These modifications were (2) removing the SP from the N-terminus and placing it at the C-terminus (SP C-term) and (3) random shuffling the amino acids of the SP at the N-terminus (SP Random). Further modifications that (4) reversed (Reverse) or (5) shuffled (Random) the entire sequence were made to compare the effect on the prediction score when the presumed common features of plant CSPs and LSPs were purposely disrupted. Both these latter modifications were expected to negatively impact SecretomeP prediction scores.

Details on how these modifications were performed via Python scripts is available online (see Prediction and modification scripts). Briefly, the amino acids for each sequence were treated as strings of text and for the reverse dataset the amino acid sequence was simply reversed. For others, SignalP 4.1 (Petersen et al., 2011) was used to predict the SP of CSPs. The predicted SP was then either automatically removed or moved to the C-terminus to generate the SP Remove and SP C-term datasets, respectively. Random shuffling (with 500 bootstrap replicates) of either the amino acids of the SP (SP Random) or the entire sequence (Random) was done using the Python random library on the respective section of the sequence and a mean prediction score from all bootstraps calculated. When comparisons to negative data were required, i.e., non-secreted proteins where no SP was predicted, the first 30 amino acids of each sequence were modified in the same manner as the SP of CSPs. For each sequence in a test set, this resulted in 1003 additional sequences.

### Prediction and Modification Scripts

The downloadable version of SecretomeP v1 was used for all predictions. The large number of permutations required for the shuffling of sequences meant that parallel processing of the prediction results was required. The downloadable version was therefore executed on multiple virtual machines using Docker containers^1^ on the NeCTAR cloud service^2^. The features used by SecretomeP rely on several other programs, hence SEG (Wootton and Federhen, 1996), PSORT II (Nakai and Horton, 1999), TMHMM 2.0c (Krogh et al., 2001) and ProP 1.0c (Duckert et al., 2004) was also installed on the same machines, as well as the optional dependency of the older version 3 of SignalP.

For the generation of modified sequence data, sequences from the data sources were modified using the steps described above (see Evaluation Data and Protein Sequence Modifications) on virtual machines with SignalP version 4 installed. The modified sequences were then split into smaller subsets for processing, and SecretomeP containers executed on these in parallel. Each set of results was collated together for analysis. The files describing the steps for the creation of these containers, as well as the scripts to modify sequences, are available at: https://github.com/lonsbio/lsp_modification_analysis.

### Accuracy Analysis

To quantify if changes in SecretomeP output scores were significant, a Student’s *T*-test between the original scores and each protein modification was calculated. Since the modified sequences are dependent on the original sequences, a paired *T*-test was used. Due to these multiple tests, an adjusted *p*-value of ≤ 0.01 was used as the significance threshold to reject the null hypothesis that a modification did not influence the SecretomeP results.

Receiver operating characteristic (ROC) curves were plotted to determine TPR and FPR for SecretomeP on plant proteins. ROC curves are useful to evaluate and visualize classifiers (Fawcett, 2006). Given a binary classifier and both positive and negative data, ROC curves are built by gradually reducing the threshold for classification and plotting each point on ROC space (as in **Figure 1**). ROC curves were plotted for pairs of positive and negative datasets (as defined in Supplementary Table S1) with the ‘scikit-learn’ package in Python. Each ROC plot represents one data set with multiple curves, with each curve representing either the original scores or one of the protein modifications. Given a random selection of a positive and negative protein from their respective datasets, the area under-the-curve (AUC) is the probability the positive protein will be classified higher than the negative protein (Fawcett, 2006).

## Results

### Mean SecretomeP Prediction Scores for Plant Classically Secreted Proteins (CSPs) Are Higher Than for Non-secreted Proteins

Various sets of proteins obtained from either WallProtDB or ASURE databases were subjected to SecretomeP prediction and the mean prediction score calculated (Supplementary Table S1). The 1983 unmodified WallProtDB proteins (Original) that were analyzed had an average prediction score of 0.707. Subsets of WallProtDB proteins, selected by protein redundancy or species, had prediction scores ranging from 0.644 to 0.746, indicating the average scores exceeded previously used thresholds of 0.5 and 0.6. For the subsets of ASURE-derived proteins, scores were lower in every case, including the sub-cellular locations with no secretory pathway involvement such as the nucleus and cytosol (0.494 and 0.446 individually, 0.479 combined). The highest score of 0.582 was obtained for the Extracellular sub-set that contains only secreted proteins. Since these scores are averaged over uneven datasets they are not conclusive, however, they do indicate that the average scores for secreted proteins are higher than for non-secreted proteins and hence that SecretomeP appears to distinguish between plant secreted and non-secreted proteins.

### SecretomeP Prediction Scores Are Influenced by the Presence of a Signal Peptide

Additional protein datasets were created through modifications to each original protein to explore the possible influence of the SP, a protein feature unique to CSPs (see Materials and Methods, Evaluation Data and Protein Sequence Modifications), and SecretomeP was applied to each modified protein dataset. It was expected that modifications to the SP only (SP Remove, SP C-term and SP Random) should not impact SecretomeP output scores whereas modifications to the entire sequence (Reverse/Random) should as protein features common to both CSPs and LSPs would be disrupted. For the full WallProtDB dataset, the mean SecretomeP output scores were lower for each modification from the Original (0.707) to: 0.522 (Reverse), 0.568 (SP Remove), 0.541 (SP C-term), 0.603 (Random), and 0.648 (SP Random). For significance, rather than compare the difference between mean scores of the entire set, the changes in scores were tested via a paired Student’s *T*-test (*p* ≤ 0.01) between original scores and the modifications (Supplementary Table S1) due to the dependence between the original and modified protein. Every modification to WallProtDB was significant, indicating that the modifications reduced the confidence with which the method assigned “secreted” status.

In contrast, the equivalent modifications to the non-secreted proteins using the nucleus and/or cytosol dataset as the exemplar, produced no substantial change in the Secretome *P* scores with means of 0.479 (Original), 0.483 (Reverse), 0.486 (SP Remove), 0.460 (SP C-term), 0.545 (Random), and 0.482 (SP Random) (Supplementary Table S1). None of the changes were significant, with the exception of some randomized sequences, which also resulted in higher average prediction scores for these non-secreted proteins. Modifications affecting the N-terminal region (where a SP is located in CSPs) significantly changed the prediction score for CSPs only, suggesting that a portion of the high predictive score for these proteins is attributable to properties of the SP, rather than any general common-feature in the mature protein.

**FIGURE 3:**
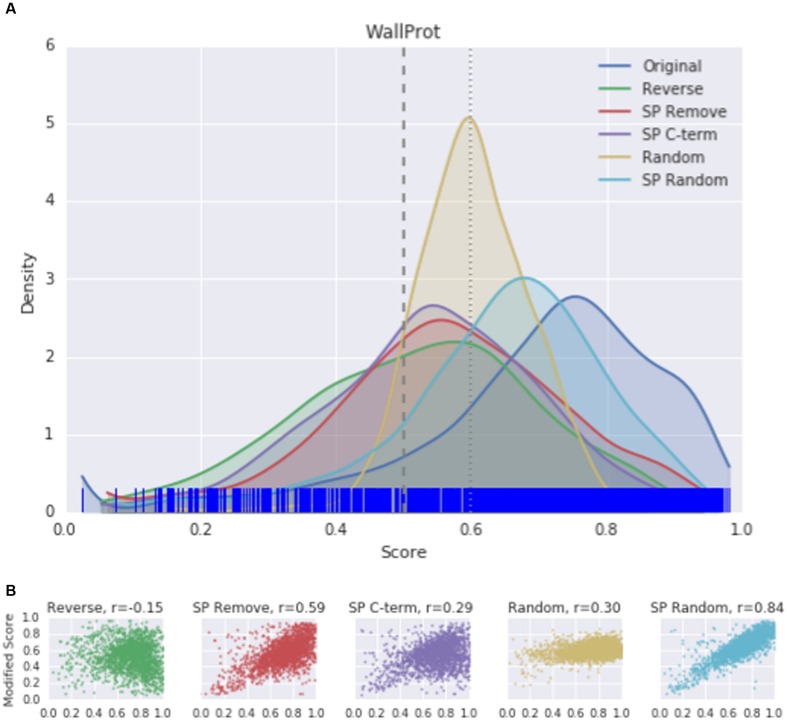
**(A)** The kernel density estimation (KDE) distributions of SecretomeP scores (v1.0) for original and modified sequences of the entire WallProtDB dataset. (Individual KDE distribution plots for sub-sets of WallProtDB are provided in Supplementary Figure S1). Rug plot marks along the *x*-axis indicate position of original scores. Vertical dashed lines at 0.5 and 0.6 represent the most common cutoff scores used in plant studies. **(B)** Correlation plots between original scores (*x*-axis) and modified scores (*y*-axis). Spearman correlation values are indicated for each modification.

Since the mean score of predictions is a summary statistic and susceptible to outliers, the distribution of scores was plotted to visualize the effect of protein modifications. A kernel density estimation (KDE) plot was used for all original proteins in each dataset, with the distribution of each modification overlaid. The curve represents the smoothed Gaussian distributions of scores. The rug plot marks along the x-axis indicating the original scores for each protein. As suggested by the significant lowering of the SecretomeP output scores, a change in the score distribution was seen in KDE plots for each protein modification compared to the original WallProtDB dataset (**Figure 3A**). Protein modifications that were both expected and not expected to alter scores did so. Sub-sets of WallProtDB, based on either plant species or maximum sequence identity threshold, exhibit the same shifts showing that this observation is not due to the composition of WallProtDB (Supplementary Figure S1).

**FIGURE 4:**
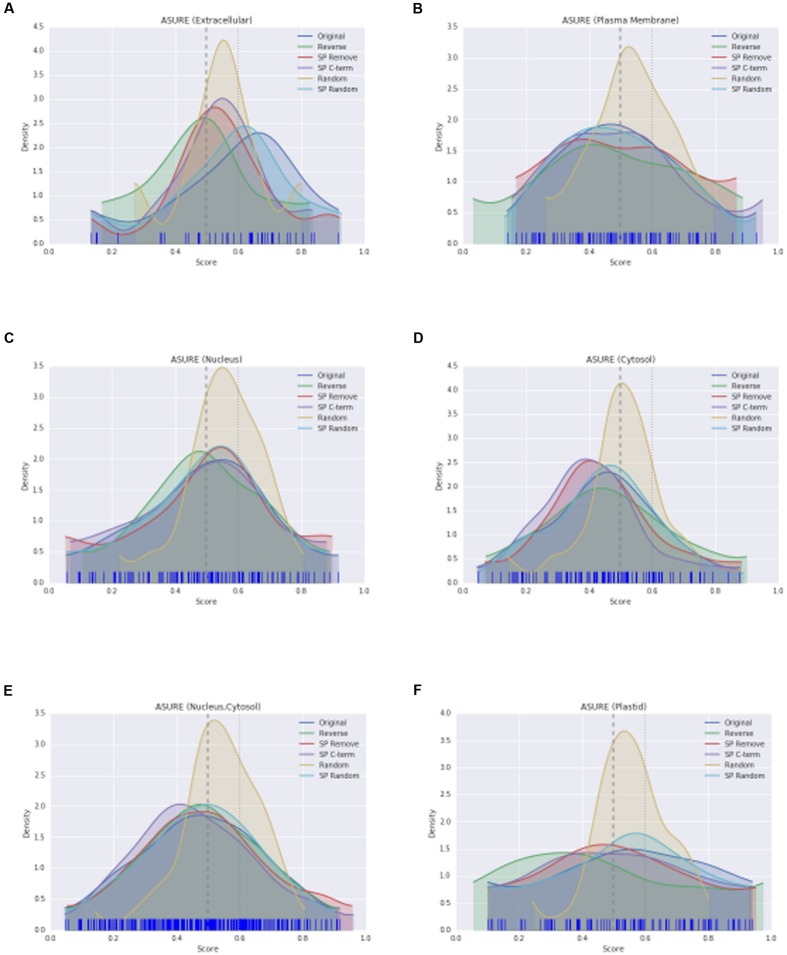
**The KDE distributions of SecretomeP scores (as described in **Figure 3A**) for protein sub-sets of ASURE.** Extracellular **(A)**, plasma membrane **(B)**, nucleus **(C)**, cytosol **(D)**, nucleus and/or cytosol **(E),** and plastid **(F)**, respectively.

The KDE plot of the modified WallProtDB dataset is distinct from the original sequences. The SP Remove, Reverse and SP C-term modifications all appear to shift the distribution toward the left, i.e., a higher density of lower scores (**Figure 3A**). Random shuffling of the SP region has the least change compared to other modifications. Given that it is the least disruptive modification to the sequence (altering the order of amino acids in only a small region of the protein that over 500 bootstraps could often resemble the original sequence), and yet it still results in a significant drop in scores, is further evidence that high prediction scores of SecretomeP are reliant on the SP. The density plot of the completely shuffled sequences (Random) were narrower with a high density of scores between 0.5 and 0.6 in all datasets. As a modification expected to alter the prediction score, this illustrates that disruption of the entire sequence does result in a change in scores, although as each score is an average of 500 bootstraps the exact range of these scores may not be informative. A more detailed investigation of the effect of SP sequence randomization and full sequence randomization on scores shows that the average value from bootstraps can hide some interesting variations (Supplementary Figure S2) but ultimately support the conclusion that the SP region can have a strong effect on SecretomeP output scores. For the ASURE dataset, the KDE plots of the secretory extracellular protein sub-set (**Figure 4A**) exhibit a similar pattern with a shift to lower scores. The mean scores did not quite reach a *p*-value to reject the common-feature hypothesis (Supplementary Table S1), likely due to the smaller sample size, however, these data suggest the same reliance of output scores on the SP as seen in the full WallProtDB dataset.

This effect of a shift when the SP region is altered is not seen for the non-secretory sub-sets of ASURE (nucleus, cytosol, nucleus and/or cytosol, plastid) proteins (Supplementary Table S1). Apart from the Random modification, each modification has a similar distribution to the original scores (**Figures 4C–E**) even though the equivalent to the SP region of the sequence was altered in the same way. The ASURE sub-sets defined as neutral also showed the same change in Random modification scores (**Figures 4B,F**). This contrast between the positive and negative datasets, and together with the significance tests and changes in mean scores demonstrate the influence of the SP on SecretomeP output scores and diminishes the likelihood that LSPs lacking this feature will be accurately predicted as “secreted.”

### Correlation between Modified and Original Sequences Differs between Secreted and Non-Secreted Proteins

To explore whether high-scoring proteins remain high-scoring after sequence modification, the Spearman correlation coefficient was calculated between the SecretomeP scores obtained from the unmodified data and the scores following sequence modification. The correlation between original score and each modification of the WallProtDB dataset is shown in **Figure 3B**. Shuffling the SP (SP Random) was least divergent at ρ = 0.84. The most divergent change was reversing the sequence, which showed a weak negative correlation. The deviation from the diagonal shows that some modifications largely dropped scores (bottom-right of correlation plot), although some improved the prediction score from low to high (top-left of correlation plot). Of note is the relatively high correlation for the SP Remove modification. The sub-sets of WallProtDB overall show consistent results with similar levels of correlation indicating conclusions drawn from these correlations holds across the plant species included in WallProtDB (Supplementary Table S2).

**FIGURE 5:**
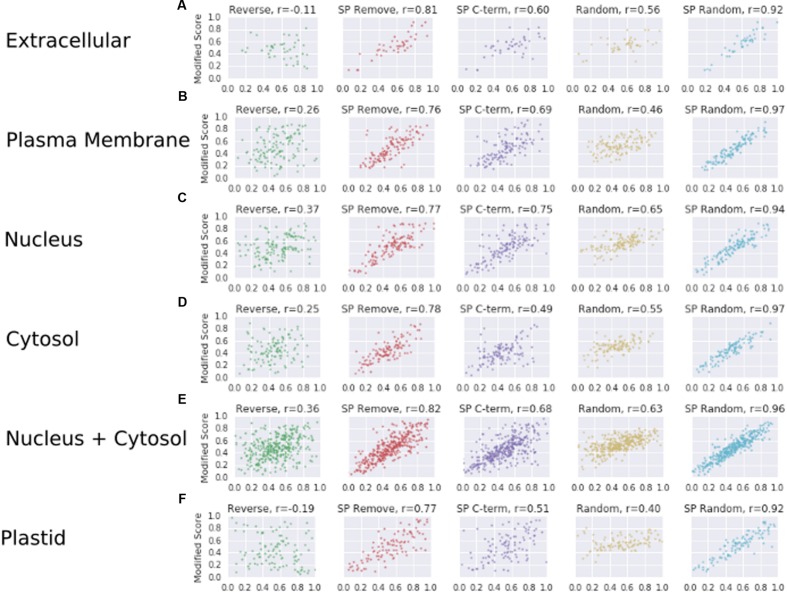
**The correlation scores (as described in **Figure 3B**) for protein sub-sets of ASURE.** Extracellular **(A)**, plasma membrane **(B)**, nucleus **(C)**, cytosol **(D)**, nucleus and/or cytosol **(E)**, and plastid **(F)**, respectively.

Correlations for the ASURE subsets are also informative (**Figure 5**). The relative positive correlation value for each modification is maintained from the WallProtDB analysis, though mostly higher values were obtained (compare **Figure 5** with **Figure 3B**, Supplementary Table S2). The exception was the Reverse modifications, which showed a small positive correlation, particularly for nucleus, cytosol and the combined sets, and a negative correlation for the WallProtDB and ASURE (Extracellular) sub-sets. This modification does not rely on a substitute SP region, and the difference in scores between positive and negative data suggests that the weak negative correlation found in WallProtDB and ASURE (Extracellular) is a feature of SP-containing proteins.

Taking into account the combination of different SecretomeP output score means, shifted distributions and correlation patterns across WallProtDB and ASURE sub-cellular organelle protein sub-sets, it is reasonable to conclude that: (1) the scores of plant CSPs from SecretomeP are influenced by the presence of a SP; (2) unmodified SP sequences are a poor proxy for LSPs; and (3) the SP Remove modification has the highest correlation with original scores (except for those involving any random shuffling) across both positive and negative data and largely produces a lower score than the original sequence, thereby making it the most suitable proxy to use when comparing the performance of SecretomeP.

### SecretomeP Performs Marginally Better Than Chance on Plant Proteins

**FIGURE 6:**
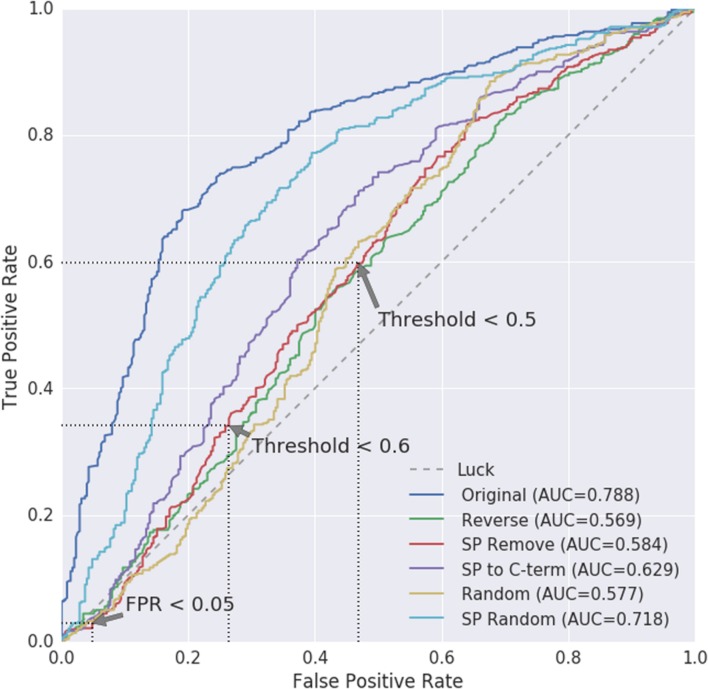
**Receiver operating characteristic (ROC) curves for positive WallProtDB *Arabidopsis* proteins vs. the negative class of ASURE proteins from the nucleus and/or cytosol.** Area Under Curve (AUC) values are shown for each protein modification. The AUC for random (pure chance/luck) is 0.5. As the threshold for classification is reduced, the true positive (TPR) and false positive (FPR) rates are mapped to the axes, giving visual insight into how the classifier balances true and false positives and which thresholds might be considered most appropriate. The preferred path of an ROC is toward the upper left hand corner signifying a high true positive rate and low false positive rate. Three points on the SP Remove curve are annotated corresponding to when either the FPR is <0.05 or threshold is set to 0.5 or 0.6. The exact values are listed in Supplementary Table S3.

WallProtDB contains many similar sequences. Therefore, to be able to compare against the *Arabidopsis* negative data in an unbiased manner, we used the *Arabidopsis* WallProtDB proteins and the ASURE (nucleus and/or cytosol) sub-set to create ROC curves comparing modified positive to modified negative datasets (**Figure 6**). The SP Remove modification is used to infer the TPR and FPR scores, although the results from other modifications are also shown.

The path of the SP Remove ROC curve shows the TPR and FPR are similar and near to the rate equivalent of “luck/chance.” The stark difference between the ROC curve based on the original sequences (dark blue) and protein sequences where the SP has been removed (SP Remove; red) confirms that the ability of SecretomeP to distinguish between secretory and non-secretory plant proteins is largely influenced by the presence of the SP in CSPs. Therefore, the curve for the SP Remove sequences is the more accurate for evaluating the use of SecretomeP to predict LSPs.

Three points of interest on the SP Remove curve are annotated with dotted lines in **Figure 6**. Each corresponds with the nearest discrete points on the ROC curve corresponding to where values of either the FPR or cut-off threshold are fixed, the exact values of which are shown for all modifications in Supplementary Table S3. Firstly, the threshold and subsequent TPR are identified when the FPR is set to be ≤0.05 (Supplementary Table S3A). For SP Remove this results in a threshold of 0.860 which leads to a TPR of 0.029 for a FPR of 0.048, indicating more false predictions than true.

Secondly, we note the TPR and FPR for thresholds around 0.5–0.6, which are the most commonly used thresholds for plant proteins (Supplementary Table S3B). These two points have higher TPR at the cost of higher FPR: the threshold near 0.6 would improve the TPR to 0.341, however, at the cost of 0.264 FPR. Further reduction to a 0.5 cut-off results in a TPR of 0.598 with a 0.469 FPR (**Figure 6**).

If the maximum area under the curve (AUC) of 0.788 and upper-left curve trajectory of the Original scores represented a realistic evaluation, then performance on plant proteins would be fair. However, the shift of the curve back toward the diagonal as soon as the SP is removed shows how dependent this predictive power is on the SP, as the AUC decreases to 0.584, and performance is poor at higher thresholds. There is no reasonable trade-off between error rates; regardless of where the threshold is drawn, the true positive and false positive rates are roughly equivalent, indicating prediction is effectively equal to random chance.

## Discussion

### Common-Features between CSPs and LSPs in Plants Are Not Captured in SecretomeP

It is difficult to assess the accuracy of a prediction tool without both positive and negative examples of the prediction target of interest. In order to evaluate how well an *in silico* method predicts LSPs, the best kind of positive data would be experimentally confirmed LSPs, but unfortunately there are very few known plant LSPs and only one protein with biochemical validation (Pinedo et al., 2012). If we accept the common feature hypothesis that underlies SecretomeP prediction, then CSPs are the next best option. For LSPs in plant studies, SecretomeP has previously been estimated to have a 0.6 TPR by assuming all proteins found in a study are positive examples, without considering negative examples. Therefore in this current study, we attempted to overcome this difficulty of assessment by virtue of the common-feature hypothesis behind SecretomeP and the expectation that performance for CSPs is informative if the hypothesis is true. Since modifications intended to disturb the SP of a sequence have a significant effect on the prediction output of positive data and not on negative data (Supplementary Table S1), we concluded that modified sequences were required to evaluate predictions based on these common features. This evaluation revealed that performance, when the FPR is limited to 0.05, resulted in true positive predictions that were not better than chance alone. Previous suggestions that SecretomeP is not well suited to plants (Agrawal et al., 2010; Ding et al., 2012; Albenne et al., 2013) were confirmed, and the estimated accuracy is so low that its use should be avoided for plant proteins. Furthermore, previous use of SecretomeP to predict putative LSPs on plant proteins must be re-evaluated in light of our findings.

### Effects of Hydrophobic Regions

WallProtDB includes some proteins from the plasma membrane involved in cell wall metabolism, such as cellulose synthase (CESA). Under the common-feature hypothesis, it is reasonable to include these in the positive dataset given the adjacency to the extracellular space, since plasma membrane-associated proteins should have some features suitable to or related to the extracellular space and would be expected to traffic through the ER/Golgi compartments. We do not consider their inclusion in WallProtDB to alter the conclusions, since the ROC results for the balanced ASURE tests are consistent with poor performance on other data.

Transmembrane domains were also excluded from the mammalian training set of SecretomeP, although the output of the TMHMM predictor was included as a candidate feature and found to be amongst the most predictive when the features were selected. Because of the ‘black box’ nature of SecretomeP’s internal workings, the exact interpretation of TMHMM outputs in determining mammalian LSPs is unknown. As noted when published, TMHMM misclassifies about 20% of SPs as helices in eukaryotes (Krogh et al., 2001). The original training set featured no SPs or transmembrane helices and so from the influence of SPs on plant CSP scores, we suggest that the presence of an amino acid sequence with similar hydrophobic properties is taken as a positive predictor of secretion. This reliance on TMHMM to identify such regions could be one of the causes for the difference in prediction scores between CSPs and truncated versions without the SP region. The inclusion of other general tools, such as PSORT II may also detect these features of CSPs and boost the prediction scores.

### Choice of Threshold

Our conclusions are drawn directly from the available data in WallProtDB and ASURE, and the selection of which sub-sets of both datasets to use for ROC analysis. As such the choice of data determines the precise TPR, FPR and threshold results. Modifications were made to both positive and negative data to create ROC curves (**Figure 6**), though comparisons between modified positive data to unmodified negative data produce similar results (Supplementary Figure S3). The *Arabidopsis* sub-set of WallProtDB and ASURE (nucleus and/or cytosol) were selected due to their size and clear exclusion from the secretory pathway and extracellular destination. Broadly, however, the results hold even if a more balanced yet smaller set, such as using ASURE for both positive and negative data is selected or additional sub-cellular locations are included as negative data (Supplementary Figures S4 and S5). The trend of the ROC curve of partial sequences toward random continues, and although the AUC is higher, there are only 42 positive proteins in the ASURE (Extracellular) sub-set as comparison, and thus it lacks power. Although the results are specific to *Arabidopsis*, the use of a general mammalian tool on plant proteins pre-supposes that the features are shared between all mammals and plants. Poor performance on *Arabidopsis* does not rule out better performance on other plant proteins, but it would require that the conserved features are poor on *Arabidopsis* only which is unlikely given the high degree of conservation of proteins across species.

The previous thresholds used for SecretomeP in the plant literature vary. Albenne et al. (2013) indicate a small number of *Arabidopsis* proteins exceed the threshold, but don’t equivocally state what threshold they used. Jamet et al. (2008) use 0.6, yet Cheng et al. (2009) use 0.5 in their finding of 0.6 TPR based on 37 LSPs they assumed were genuine. This TPR drops to 0.3 when using a more stringent 0.6 threshold (Supplementary Table S4). The output of the web server version for SecretomeP^3^ previously indicated 0.5 should be used as a cutoff (accessed August 2015) which matches the more recent bacterial version. Currently (accessed April 2016) the server indicates both 0.5 and 0.6 thresholds for bacterial and mammalian proteins, respectively, in line with the published results. This change could contribute to the multiple values used, however, since SecretomeP is not explicitly listed as a tool for plant proteins, which threshold score to use to match the expected sensitivity and specificity it provides when applied to plant proteins is lacking. The pervasiveness of a precise value in the literature, however, indicates that it may be useful to use the thresholds in Supplementary Table S3 to recommend a different value with a known FPR, which could be preferable to the status quo.

The original cross-validation estimate of SecretomeP sensitivity (TPR) was 0.4 when FPR is 0.05 and it was acknowledged that this would not classify all LSP proteins without using a much lower threshold, thereby incurring a high cost of false positives (Bendtsen et al., 2004). From Supplementary Table S3, the upper bound of unmodified sequences would indicate that the TPR around 0.276 occurs for a threshold of 0.794, and that estimates of the effect of the SP will only reduce this. Given our results showing the reliance on the SP when using CSPs as proxy, and based on the performance of SecretomeP on modified CSPs with the SP Removed, controlling the FPR to match the mammalian version at below 0.05 requires the threshold to be 0.86 and the subsequent TPR is 0.029 (Supplementary Table S3A). These values represent the values at which we estimate SecretomeP can predict based on conserved features with some degree of certainty and therefore the use of SecretomeP on plants does not appear justified. Although it would represent an improvement over current usage and match the FPR of the original program, excluding proteins on this basis would mean losing the vast majority of true positives from any proteome; the expectation would be that more false-positives would still be identified than true secreted proteins.

### Upper and Lower Limits on Performance

The discrepancy between scores for original and truncated CSPs are evidence against common features of plant proteins being recognized. The theory behind SecretomeP is that the mechanism of secretion should not be captured, and that other shared aspects of proteins that make it suitable for secretion are what predictions are based on.

There remains the possibility, however, that shared features in plants between CSPs and LSPs could also resemble the SP mechanism (e.g., the similar hydrophobic properties between SPs and GPI anchor regions) and that performance on true LSPs may be similar to the original sequence results seen in this study, if such features are highly predictive. Under this scenario, the ROC curve for the original sequence could represent an approximate upper bound on the accuracy, and the SP Remove the lower. This would still not make SecretomeP suitable for plant proteins, as the predictions would not be consistent with its original hypothesis based on common-features between CSPs and LSPs, but rather on similarities between the SP mechanism and LSP specific features. This scenario would also not account for reduced scores for CSPs, as these features should exist in addition to the SP region if they are truly conserved.

This illustrates one difficulty with the mammalian trained common-feature hypothesis being transferred to plants. This work shows that in plants the secretion mechanism is influential to the prediction score, but masks the true performance value, which is likely to be somewhere between the Original and SP Removed sequence curves (**Figure 6**). Future work using the common-feature hypothesis in plants should take these types of protein regions into account, namely if sequences are modified for training a tool, then during development they must be checked to ensure both modified and unmodified sequences have a similar accuracy so that common-features are captured rather than secretion mechanisms.

### New Prediction Tools and Approaches Are Required

SecretomeP was not trained on plant proteins. There is no claim made in either the original publication or in the currently available web service that SecretomeP has any applicability to plants. Although version 1.0 could be considered ‘eukaryotic’ by inference from the text available on the archived website^4^, the abstract clearly states the method is for mammalian secretory proteins. The current version offers modes of operation for mammals and bacteria only. Applying SecretomeP to plant protein data implies a chain of assumptions that the features shared between mammalian CSPs and LSPs are conserved and also shared with plant CSPs and LSPs. Our results show that these assumptions do not hold, and that observations of the accuracy in the literature are not supported. While it is tempting to reduce a large dataset into something manageable for further analysis the current study has shown that the predictive power of SecretomeP on plant proteins is such that true-positives will be found at a rate close to the false-positive rate, with marginal improvement over random selection of classes.

Instead of SecretomeP being ‘better than nothing,’ we propose that it is actually ‘worse than nothing’ so it is better to do nothing than to use it on plant proteins. Independent experimental lines of evidence (biochemical/cell biological) are required confirm possible LSPs, but in plants, SecretomeP should not be used to identify putative LSPs. Depending on the consequences of either false positives or false negatives in a proteomic study, strategies to accept all proteins found, or reject those without a SP, are advised. A filtering strategy should not be used until a plant-specific tool is developed.

## Author Contributions

AL devised and performed the analyses, in consultation with MoD, MeD, and AB. All Authors contributed to data interpretation and writing of the manuscript.

## Conflict of Interest Statement

The authors declare that the research was conducted in the absence of any commercial or financial relationships that could be construed as a potential conflict of interest.

## Footnotes

1 http://docker.com

2 http://nectar.org.au

3 http://www.cbs.dtu.dk/services/SecretomeP/

4 http://www.cbs.dtu.dk/services/SecretomeP-1.0/
