# Supplementary material for: Better Than Nothing? Limitations of the Prediction Tool SecretomeP in the Search for Leaderless Secretory Proteins (LSPs) in Plants
Source: Front Plant Sci. 2016 Sep 27;7:1451. doi: 10.3389/fpls.2016.01451 (PMC5037178; doi:10.3389/fpls.2016.01451)
Supplement: Supplementary file 1 [file Data_Sheet_1.DOCX]

Supplementary Material

Better than nothing? Limitations of the prediction tool SecretomeP in the search for leaderless secretory proteins (LSPs) in plants.

Andrew Lonsdale^1^, Melissa J Davis^2^, Monika S Doblin^1^ and Antony Bacic^1,*^

*** Correspondence:** Antony Bacic: abacic@unimelb.edu.au

# Supplementary Figures and Tables

**Supplementary Table S1.** Mean SecretomeP output scores for the all positive and negative input data from WallProtDB and ASURE databases. Shaded scores indicate statistically significant changes based on a paired student T-test between modified and unmodified sequences which considers the mean of the differences in scores is due to the dependence between original and modified protein ( *p ≤ 0.01, **p ≤ 0.001 and ***p ≤ 0.0001).

| **Data** | **#^1^** | **Data^2^** | **Original** | **Reverse** | **SP Remove** | **SP c-term** | **Random** | **SP Random** |
| --- | --- | --- | --- | --- | --- | --- | --- | --- |
| **WallProt** | 1983 | Positive | 0.707 | 0.522^***^ | 0.568^***^ | 0.541^***^ | 0.603^***^ | 0.648^***^ |
| WallProt (30% maximum sequence identity) | 278 | Positive | 0.644 | 0.499^***^ | 0.542^***^ | 0.517^***^ | 0.580^***^ | 0.598^***^ |
| WallProt (*Arabidopsis*) | 522 | Positive | 0.683 | 0.525^***^ | 0.536^***^ | 0.534^***^ | 0.573^***^ | 0.614^***^ |
| WallProt (excl. *Arabidopsis*) | 1461 | Positive | 0.716 | 0.521^***^ | 0.579^***^ | 0.544^***^ | 0.615^***^ | 0.660^***^ |
| WallProt (Rice) | 208 | Positive | 0.746 | 0.483^***^ | 0.591^***^ | 0.529^***^ | 0.616^***^ | 0.688^***^ |
| WallProt (*Brachypodium*) | 358 | Positive | 0.728 | 0.471^***^ | 0.556^***^ | 0.521^***^ | 0.605^***^ | 0.665^***^ |
| **ASURE Proteins (all)** | 975 (73 dual) | Mixed | 0.503 | 0.480^**^ | 0.492^**^ | 0.477^***^ | 0.550^***^ | 0.499 |
| ASURE (Nucleus) | 130 | Negative | 0.494 | 0.503 | 0.517 | 0.492 | 0.565^***^ | 0.499 |
| ASURE (Cytosol) | 112 | Negative | 0.446 | 0.452 | 0.445 | 0.412 | 0.507^***^ | 0.445 |
| ASURE (Nucleus + Cytosol) | 352 (110 dual) | Negative | 0.479 | 0.483 | 0.486 | 0.460 | 0.545^***^ | 0.482 |
| ASURE (Mitochondrion) | 70 | Neutral | 0.578 | 0.520 | 0.555 | 0.537 | 0.584 | 0.571 |
| ASURE (Peroxisome) | 12 | Neutral | 0.524 | 0.450 | 0.464 | 0.393 | 0.555 | 0.506 |
| ASURE (Plastid) | 103 | Neutral | 0.541 | 0.437^*^ | 0.502^*^ | 0.503 | 0.565 | 0.532 |
| ASURE (Nucleus, Cytosol, Plastid, Mitochondrion, Peroxisome) | 555 (18 dual) | Mixed | 0.505 | 0.479 | 0.497 | 0.478^**^ | 0.553^***^ | 0.504 |
| ASURE (Extracellular) | 42 | Positive | 0.582 | 0.475 | 0.543 | 0.527 | 0.555 | 0.573 |
| ASURE (Plasma Membrane) | 119 | Neutral | 0.492 | 0.494 | 0.490 | 0.486 | 0.539^*^ | 0.482 |
| ASURE (Extracellular + Plasma Membrane) | 177 (16 dual) | Mixed | 0.524 | 0.490 | 0.500 | 0.495 | 0.545 | 0.511^**^ |
| ASURE (Golgi) | 73 | Neutral | 0.487 | 0.446 | 0.467 | 0.455 | 0.523 | 0.479 |
| ASURE (Vacuole) | 50 | Neutral | 0.506 | 0.521 | 0.505 | 0.503 | 0.616^*^ | 0.509 |
| ASURE (Endoplasmic Reticulum) | 32 | Neutral | 0.483 | 0.449 | 0.479 | 0.443 | 0.516 | 0.474 |
| ASURE (Endoplasmic Reticulum + Golgi) | 120 (15 dual) | Neutral | 0.487 | 0.451 | 0.475 | 0.462 | 0.529 | 0.480 |

Number of proteins in data set. Where multiple locations are included in sub-sets, they contain additional dual-located proteins excluded from individual sub-sets.

Indicates data (sub)set contain: Positive – CSPs as proxy for LSPs, Negative - non-secreted proteins, Neutral – secretory pathway or other organelles not used as negative data for ROC curves, Mixed – combinations of positive, negative and neutral data

**Supplementary Table S2.** Spearman correlation scores between original and modified sequences. Negative correlation are shaded.

| **Data** | **Reverse** | **SP Remove** | **SP C-term** | **Random** | **SP Random** |
| --- | --- | --- | --- | --- | --- |
| **WallProt** | -0.15 | 0.59 | 0.29 | 0.30 | 0.84 |
| WallProt (30% maximum sequence identity) | -0.09 | 0.65 | 0.44 | 0.42 | 0.89 |
| WallProt (Arabidopsis) | -0.13 | 0.61 | 0.44 | 0.34 | 0.82 |
| WallProt (excl. *Arabidopsis*) | -0.15 | 0.58 | 0.23 | 0.27 | 0.84 |
| WallProt (Rice) | -0.14 | 0.61 | 0.18 | 0.21 | 0.87 |
| WallProt (*Brachypodium*) | -0.22 | 0.59 | 0.27 | 0.16 | 0.87 |
| ASURE (Extracellular) | -0.11 | 0.81 | 0.60 | 0.56 | 0.92 |
| ASURE (Plastid) | -0.19 | 0.77 | 0.51 | 0.40 | 0.92 |
| ASURE (Nucleus) | 0.37 | 0.77 | 0.75 | 0.65 | 0.94 |
| ASURE (Cytosol) | 0.25 | 0.78 | 0.49 | 0.55 | 0.97 |
| ASURE (Nucleus + Cytosol) | 0.36 | 0.82 | 0.68 | 0.63 | 0.96 |
| ASURE (Plasma Membrane) | 0.26 | 0.76 | 0.69 | 0.46 | 0.97 |

**Supplementary Table S3.** Corresponding thresholds, TPR and FPR rates calculated from Figure 6 based on values where the FPR is below 0.05 (a) and using the common thresholds of 0.5 and 0.6 as found in the plant literature (b). In each, the values from the original sequences are in bold, and from the SP Remove modification in italics.

(a)

|  | TPR | FPR | Threshold |
| --- | --- | --- | --- |
| **Original** | **0.276** | **0.043** | **0.794** |
| Reverse | 0.044 | 0.045 | 0.810 |
| *SP Remove* | *0.029* | *0.048* | *0.860* |
| SP C-term | 0.036 | 0.045 | 0.817 |
| Random | 0.033 | 0.043 | 0.735 |
| SP random | 0.130 | 0.048 | 0.772 |

(b)

|  | TPR | FPR | Threshold |
| --- | --- | --- | --- |
| **Original** | **0.858** | **0.469** | **0.500** |
| **Original** | **0.753** | **0.298** | **0.600** |
| Reverse | 0.573 | 0.457 | 0.500 |
| Reverse | 0.330 | 0.281 | 0.598 |
| *SP Remove* | *0.598* | *0.469* | *0.500* |
| *SP Remove* | *0.341* | *0.264* | *0.600* |
| SP C-term | 0.617 | 0.389 | 0.500 |
| SP C-term | 0.360 | 0.236 | 0.600 |
| Random | 0.839 | 0.656 | 0.500 |
| Random | 0.347 | 0.324 | 0.600 |
| SP random | 0.814 | 0.486 | 0.499 |
| SP random | 0.634 | 0.278 | 0.599 |

**Supplementary Table S4.** Putative LSPs adapted from Table 1 in Cheng et al. (2009), with the prediction scores and classification using 0.5 and 0.6 thresholds.

| AGI number | protein name and annotation | SecretomeP Score | LSP (0.5) | LSP (0.6) |
| --- | --- | --- | --- | --- |
| AT3G16450 | Jacalin- related protein (lectin) | 0.93 | YES | YES |
| AT1G23410 | Ubiquitin extension protein | 0.85 | YES | YES |
| AT3G32980 | PER32, peroxidase | 0.79 | YES | YES |
| AT3G07390 | AIR12, extracellular matrix constituent | 0.76 | YES | YES |
| AT5G53560 | Cytochrome b5 reductase | 0.76 | YES | YES |
| AT5G18100 | CSD3, Cu/Zn superoxide dismutase | 0.74 | YES | YES |
| AT1G08830 | CSD1, Cu/Zn superoxide dismutase | 0.73 | YES | YES |
| AT4G29680 | Phosphodiesterase | 0.73 | YES | YES |
| AT3G43810 | CAM7, Calmodulin | 0.64 | YES | YES |
| AT1G11580 | Pectinesterase (methylesterase) | 0.60 | YES | YES |
| AT2G36530 | LOS2 (low expression of osmotically responsive prot.), phosphopyruvate hydratase | 0.60 | YES | YES |
| AT3G16410 | jacalin- related protein (lectin) | 0.57 | YES | NO |
| AT4G30920 | Cytosolic aminopeptidase family | 0.57 | YES | NO |
| AT2G15130 | Basic secretory protein (BSP) family protein | 0.56 | YES | NO |
| AT3G1639 | jacalin- related protein (lectin) | 0.56 | YES | NO |
| AT3G03910 | Glutamate dehydrogenase | 0.53 | YES | NO |
| AT3G08900 | RGP, Œ±‚àí1,4-glucan-protein synthase | 0.51 | YES | NO |
| AT4G30910 | Cytosolic aminopeptidase family | 0.51 | YES | NO |
| AT5G15650 | Œ±-1,4-glucan-protein synthase | 0.51 | YES | NO |
| AT5G16510 | Reversibly glycosylated polypeptide | 0.51 | YES | NO |
| AT5G18170 | GDH1, glutamate dehydrogenase | 0.51 | YES | NO |
| AT3G03780 | AtMS2, methionine synthase | 0.50 | NO | NO |
| AT3G02230 | ATRGP1, reversibly glycosylated polypeptide | 0.49 | NO | NO |
| AT4G23590 | Aminotransferase | 0.47 | NO | NO |
| AT5G07440 | Glutamate dehydrogenase | 0.47 | NO | NO |
| AT5G17920 | ATMETS, methionine synthase | 0.47 | NO | NO |
| AT2G24200 | Leucine aminopeptidase | 0.46 | NO | NO |
| AT2G36460 | Fructose-bisphosphate aldolase | 0.46 | NO | NO |
| AT1G61820 | BGLU46, Glycoside hydrolase | 0.44 | NO | NO |
| AT1G08110 | Lactoylglutathione lyase | 0.37 | NO | NO |
| AT1G78380 | GST8, glutathione transferase | 0.35 | NO | NO |
| AT5G55730 | FLA1, fasciclin-like arabinogalactan protein | 0.33 | NO | NO |
| AT3G16420 | PBP1, assists PYK10 (Œ≤-glucosidase complex) activity in pest damaged tissues. | 0.29 | NO | NO |
| AT3G16430 | Jacalin- related protein (lectin) | 0.25 | NO | NO |
| AT1G12080 | Similar to vacuolar calcium binding protein | 0.23 | NO | NO |
| AT4G25100 | FSD1, iron superoxide dismutase | 0.20 | NO | NO |
| AT4G20260 | DREPP, plasma membrane protein | 0.03 | NO | NO |

0.5 threshold = 21/37 = 0.567 TPR

0.6 threshold = 11/37 = 0.297 TPR

## Supplementary Figures

1.
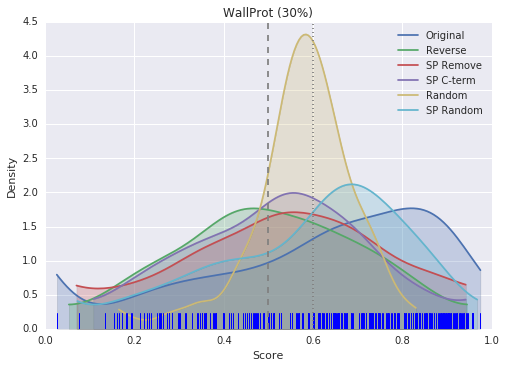
 b)
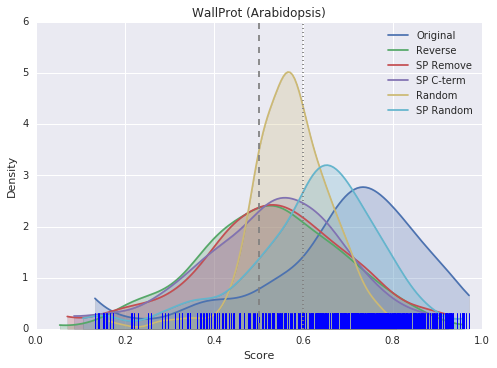


c)
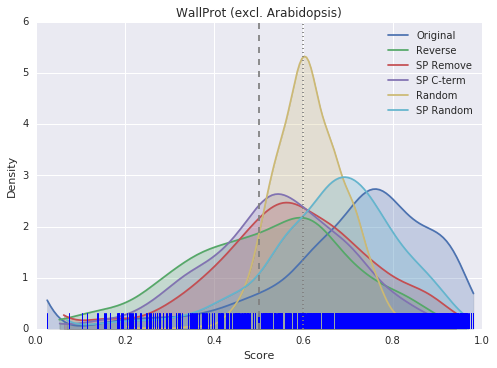
 d)
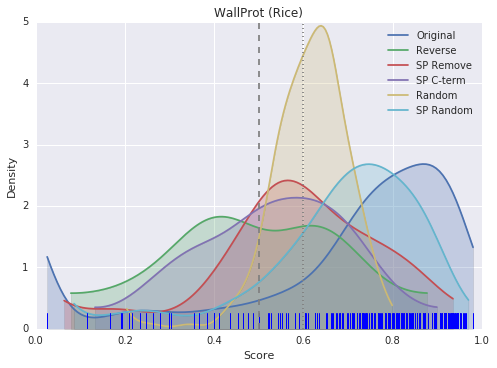


e)
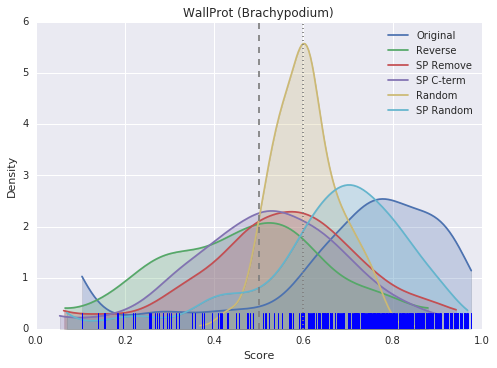


**Supplementary Figure S1.**  The KDE distributions of SecretomeP scores (as described in Figure 3a) for original and modified sequences of subsets of WallProtDB with maximum 30% sequence identity (a), *Arabidopsis* only (b), excluding *Arabidopsis* (c), rice only (d) and *Brachypodium* only (e).

##
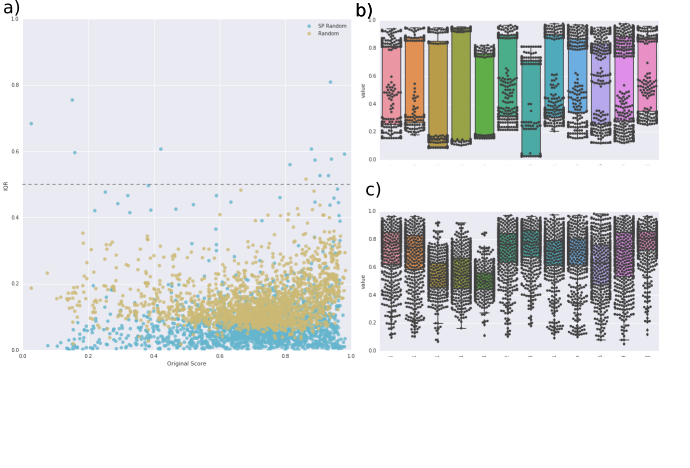


**Supplementary Figure S2.**  WallProtDB sequences with modifications involving random shuffling. The interquartile range (IQR) for each sequence from 500 bootstraps is shown in (a). The 12 proteins with IQR > 0.5 for the SP Random modification are shown in (b) as a boxplot of the individual bootstrap scores and the corresponding Random bootstrap scores for the same 12 proteins (c). In these 12 examples, modifications to the SP region tend to group into either very high or very low scores, and for the completely shuffled equivalent sequence, these extreme scores are possible but not typical.


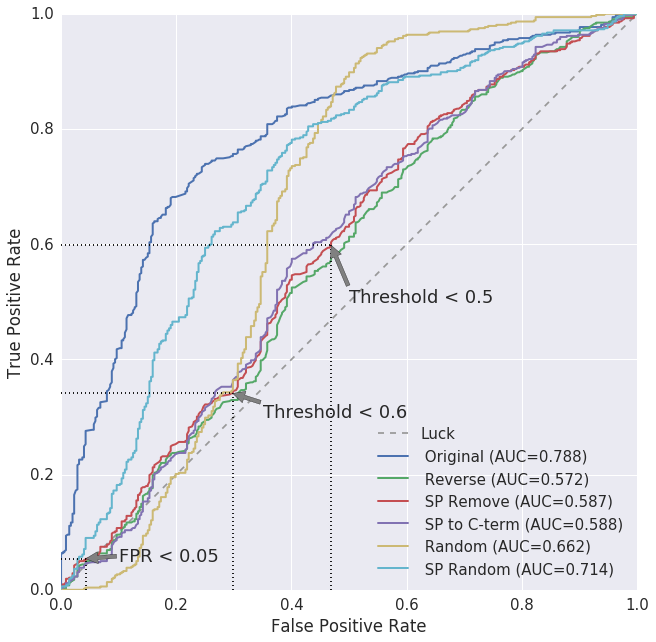


**Supplementary Figure S3:** ROC curves (as described in Figure 6) for modified WallProtDB *Arabidopsis* proteins as a positive class versus unmodified ASURE nuclear and/or cytosolic proteins as a negative class. Contrary to Figure 6, for each modification curve only the positive WallProtDB data is modified, and is compared to unmodified (Original) scores of the negative data. Three points on the SP remove curve are annotated corresponding to when either the FPR is <0.05 or threshold is set to 0.5 or 0.6.

1. **b)**


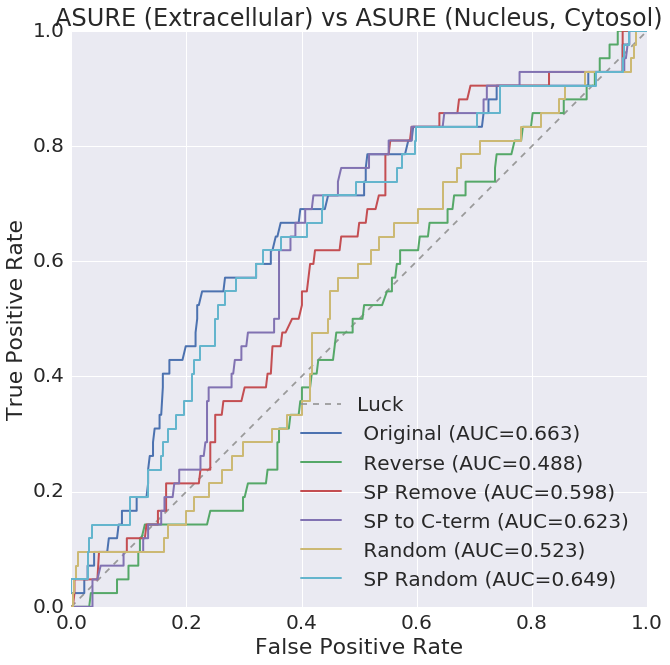

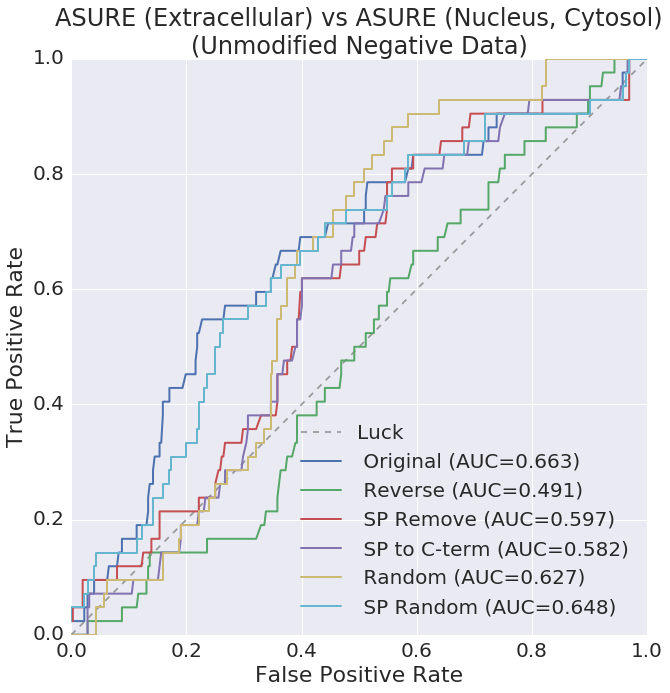


**Supplementary Figure S4:** ROC curves, constructed in the same way as Figure 6 except using modified ASURE (Extracellular) proteins as a positive class. Sub-sets of ASURE with nuclear and/or cytosolic proteins were selected as a negative data either (a) modified or (b) unmodified.

**a) b)**


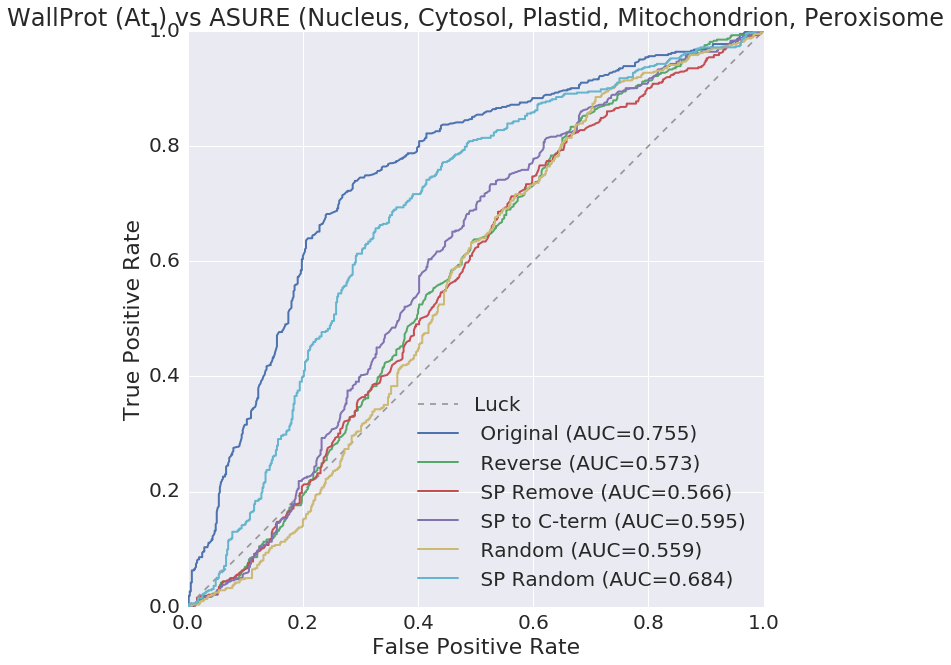

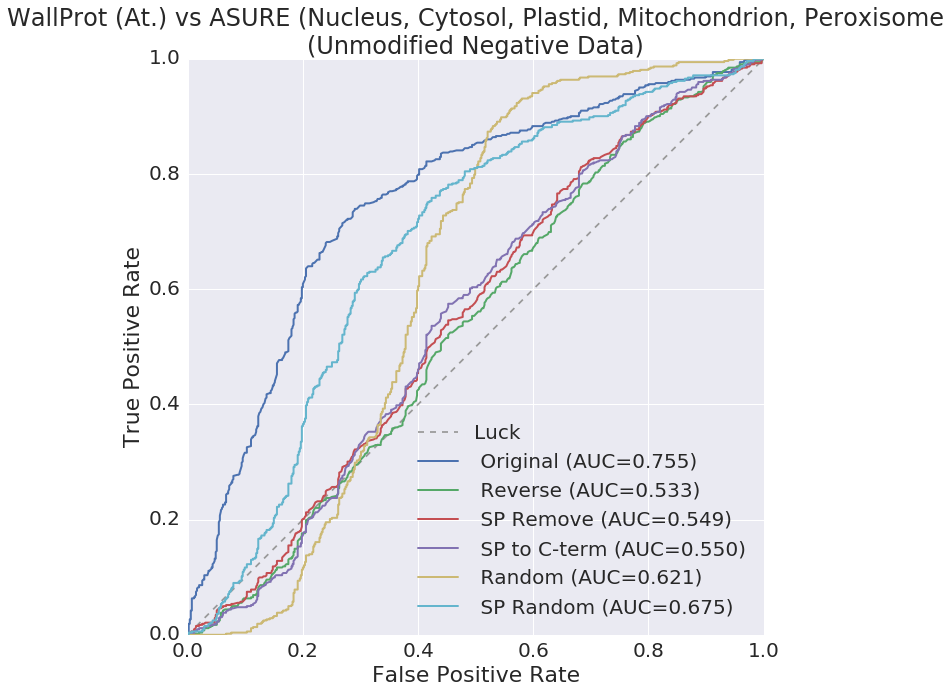


**Supplementary Figure S5:** Additional ROC curves, with *Arabidopsis* WallProtDB proteins as positive data and an expanded negative data set of ASURE nuclear, cytosolic, plastid, mitochondrion and plastid proteins (a) modified (as per Figure 6) or (b) unmodified (as per Supplementary Figure 3), respectively.
